# Results of tricuspid valve reconstruction using a semirigid ring or a flexible band

**DOI:** 10.1186/1749-8090-10-S1-A317

**Published:** 2015-12-16

**Authors:** Ivan Aleksic, Jörg Hoffmann, Michal Glanowski, Ina Schade, Sebastian P Sommer, Marcus Leistner, Armin Gorski, Rainer G Leyh

**Affiliations:** 1 Department of Thoracic and Cardiovascular Surgery, University Hospital Würzburg, Würzburg, 97080, Germany

## Background/Introduction

Current concepts for tricuspid valve surgery recommend concomitant tricuspid valve reconstruction (TVR) utilizing annuloplasty devices over simple suture annuloplasty.

## Aims/Objectives

It is unclear if flexible bands offer the same long-term results like rigid or semi-rigid rings. We aimed to evaluate possible outcome differences between the flexible SJM Tailor Band (FB group) and the Carpentier-Edwards rigid MC3-Ring (RR group).

## Method

We retrospectively evaluated 141 patients undergoing tricuspid valve reconstruction (TVR) with the MC3-ring or the Tailor Band or suture annuloplasty between 01/11 and 12/13. Demographic variables, intraoperative parameters, pre- and postoperative echocardiographic studies were analyzed. A total of 108 pts undergoing TVR formed the RR group and 20 the FB group. Patients treated with suture annuloplasty or other devices were excluded.

## Results

Age, gender, preop. Euroscore II and severity of tricuspid regurgitation (TR) did not differ among groups. Intraoperative parameters (procedure time, cardiopulmonary bypass and cross-clamp time) were also similar. Long-term mortality with a median follow-up of 1361d (range: 854-1900d) was similar, too. Postoperative echocardiographic follow-up revealed a trend for more severe TR after reconstruction with the band than with the rigid ring (p = .064, Mann-Whitney-U). Subgroup analysis of patients undergoing mitral valve plus concomitant tricuspid valve reconstruction demonstrated more moderate TR in the FB group (Table 1).

**Table 1:** Group/degree of TI TI grade I TI grade II p = 0.043 (Mann-Whitney-U)

RR group (N = 38)	25	13
FB group (N = 9)	2	7

## Discussion/Conclusion

Tricuspid valve reconstruction with the rigid MC3 ring yields a higher rate of just trace tricuspid regurgitation than with a flexible band in patients undergoing concomitant mitral valve surgery. A trend towards more trace tricuspid regurgitation was observed in the overall study cohort. Longer follow-up or even more desirable a multi-center study is warranted to determine if usage of flexible bands will lead to more reoperations or more overt right-sided heart failure than rigid rings.

